# Inverse cascade of the vortical structures near the contact line of evaporating sessile droplets

**DOI:** 10.1038/s41598-019-43289-1

**Published:** 2019-05-01

**Authors:** Abbas Ghasemi, Burak Ahmet Tuna, Xianguo Li

**Affiliations:** Department of Mechanical and Mechatronics Engineering, University of Waterloo 200 University Avenue West, Waterloo Ontario, N2L 3G1 Canada

**Keywords:** Mechanical engineering, Fluid dynamics

## Abstract

Microscopic imaging as well as the particle image velocimetry (PIV) are carried out to evaluate the concentration, velocity and vorticity fields near the contact line of the nano-particles-laden evaporating sessile droplets. After the onset of the linear thermocapillary instabilities due to the Marangoni perturbations, the non-linear state sets in and the micro-scale jet-like vortex structures are ejected from the contact line towards the center of the droplet. Afterwards, the jet-like vortical structures expand in the spanwise directions and start to interact with the neighbouring structures. Two types of the inverse cascade mechanisms are found to occur. In the first kind, the vortices of the similar length scale merge and continuously produce larger vortices and corresponding wavelength growth. The second inverse cascade mechanism takes place due to the entrainment of the smaller vortices into the larger structures. Both inverse cascade processes are identified as the continuous feeding of the kinetic energy from the smaller scales to the larger scales. For individual micro-jets the velocity field characterizes the jet-like vortex structures ejected from the contact line towards the droplet center opposing the bulk flow from the center towards the contact line. In addition, the vorticity field overlaid by the velocity streamlines identify the sense of rotation of the low pressure zones on either side of the micro-jet as well as the high pressure stagnation point at the tip.

## Introduction

Present communication reports the observation of the co-occurrence of two remarkable phenomena which have fascinated many researchers working in the fields of the physical sciences and thermofluids. The first phenomenon is the onset and growth of the thermocapillary instabilities near the contact line of the nano-particle-laden evaporating sessile droplets, and the second mechanism is the concept of the inverse energy cascade of the associated vortical structures.

Evaporation of a liquid drop seems to be as ordinary as spotting a deposited coffee stain on our kitchen table when all the liquid is vaporized^1^. For a blowfly however, the evaporation process of a droplet is as pleasant as cooling off in a hot summer day^2^. In fact, the underlying physics associated with the evaporation of the nanoparticle-laden sessile droplet are very complicated due to the interplay of numerous co-occurring processes. Evaporation of the droplets play important roles in a wide range of applications such as the ink jet printing^3^, DNA chains alignment within the drying liquid crystals^4^, red blood cell dried patterns^5^, bacterial motion^6^, cellulose nanocrystal^7^, genomic DNA in the presence of hematite nano-particles^8^ and the self-cleaning of environmental dust^9^. From a fundamental point of view, parameters such as the onset of the thermocapillary instabilities arising during the heat/mass transfer^10,11^, the shape of the colloidal particles^12^, substrate heat transfer characteristics^10^, ambient temperature and relative humidity^13^, substrate hydrophobicity^14^, and the microscopic surface roughness^15^ may either result in the formation of a coffee-ring or a homogeneous deposition by altering the internal fluid flow of the sessile droplet. Therefore, there have been attempts to use active control strategies such as the acoustic suppression^16^ or the laser-induced differential evaporation^17^ in order to obtain the desirable outcome from the evaporation process. Furthermore, the internal fluid flow of the evaporating sessile droplets and the consequent deposited patterns can also be interpreted through the study of the hydrothermal theory of stability. Under certain conditions, the perturbations in different parameters such as the surface tension, concentration, temperature, pressure and velocity may grow into the hydrothermal instabilities^11^. Namely, the Rayleigh-Benard-Marangoni types of instabilities are found to alter the convective heat/mass transfer regimes inside the evaporating sessile droplets^18,19^.

Regarding the energy cascade phenomenon, recent images of the Jupiter’s turbulent weather layer demonstrate a fascinating visualization of the both forward and inverse mechanisms^20^. What makes the mechanism of the energy cascade impressive is the fact that it can occur from the micro-scales to the astrophysical scales. The vortical structures of different scales, or the so-called eddies, seem to co-exist and interact through the momentum transfer^20^. Such eddies are predominantly observed in the nature and continuously exchange kinetic energy within the variety of fluid flows. In the forward (direct) energy cascade, kinetic energy is transferred from the largest (integral) structures to the intermediate (Taylor) scales and eventually to the smallest (Kolmogorov) scales where the kinetic energy is eventually dissipated to heat due to the dominance of the viscous effect^21^. Conversely for the inverse cascade phenomenon, the kinetic energy is frequently fed to the larger scales by the small structures^22–26^. The inverse cascade mechanism is a vibrant ongoing research field which is not only limited to the above rotational and turbulent flows or the transition of a toroidal plasma^27^, but is also investigated within the realm of the irrotational/inviscid super-fluids^28^.

Above survey through the literature provides interesting information about the dynamics of the sessile droplet evaporation as well as the inverse energy cascade phenomenon. While the two topics are generally investigated independently by the researchers of various backgrounds, the present study is motivated to study their co-occurrence. To that end, microscopic imaging as well as the particle image velocimetry (PIV) measurements are conducted to obtain the concentration, velocity and vorticity fields near the contact line of the nano-particles-laden evaporating sessile droplets. The study focuses on the onset of the linear thermocapillary instabilities, their transition into the non-linear state and the formation of the vortical structures. Moreover, the occurrence of the inverse energy cascade associated with the micro-scale vortex structures is described.

## Results

Prior to the detailed discussion of the results in following sections, it might be noted that the reported instantaneous phenomena have been selected out of measurements on 47 different droplet tests. For each droplet more than 500 images at different time instants during its drying process have been captured. The remarkable micro scale vortex dynamics phenomena have been repeatedly observed for all the 47 cases studied in the present investigation. Since the reported phenomena take place due to the onset of the instabilities with a “transient base flow”, the flow characteristics and their evolutions are described first, followed by quantitative analysis of the similar results obtained.

**Figure 1:**
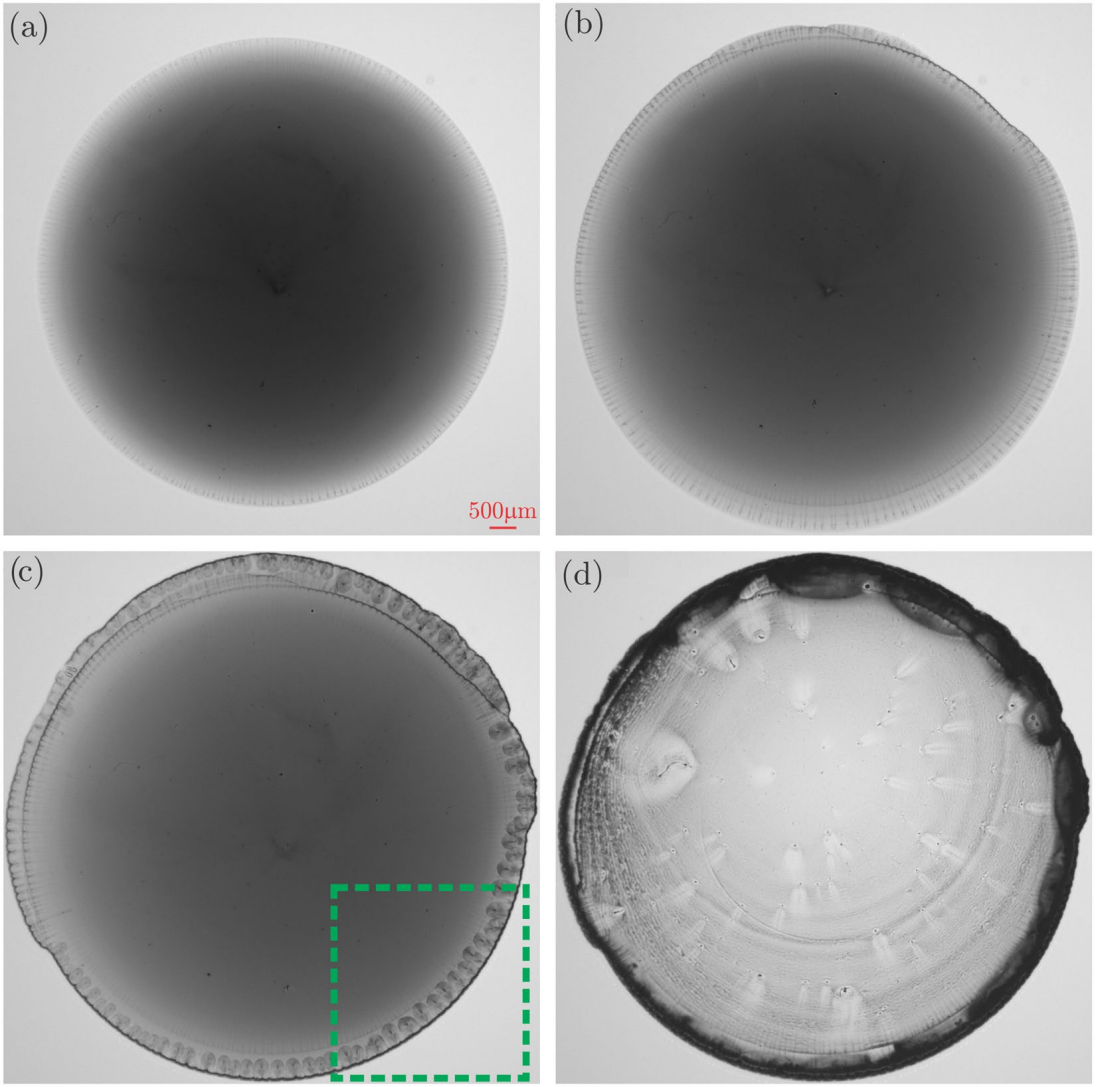
Representative raw images acquired from the present microscopy at arbitrary time instants: (**a**) Deposited droplet at its maximum radial spread; (**b**) Streak lines aligned with the radius of the circular droplet; (**c**) Micro-scale jet-like vortex structures ejected from the contact line towards the center; (**d**) Deposited coffee ring after the end of evaporation. The dashed square box represents a typical interrogation area defined for the data acquisition/processing.

In Fig. 1, representative raw images acquired from the present microscopy are demonstrated for arbitrary instants during the evaporation of the sessile droplet. In Fig. 1a, the deposited droplet reaches its maximum radial spread while the particles are rather uniformly distributed. Later in Fig. 1b, the near contact line edges of the droplet demonstrate streak lines aligned with the radius of the circular droplet. The streak lines are replaced by the micro-scale jet-like structures as shown in Fig. 1c. Finally after the entire liquid is vaporized, the deposited nano-particles demonstrate the so-called coffee ring pattern as shown in Fig. 1d. The coffee ring effect is attributed to the darker (higher particle concentration) region of the dried pattern remaining near the contact line of the evaporating sessile droplet. As described earlier, such complicated thermo-physical phenomena can be studied from different angles. Therefore, the present study is focused on explaining the observation of a remarkable phenomenon taking place near the contact line of the evaporating sessile droplet. Accordingly, the data acquisition/processing is conducted for the interrogation areas similar to the dashed square box shown in Fig. 1c. In the following sections, the relevant physical phenomena are described in the three stages itemized below:

- Stage I: Onset of the linear instability, non-linear growth and micro-jet formation
- Stage II: Micro-jet pairing
- Stage III: Inverse cascade phenomenon

After describing the three stages above, the velocity and vorticity information in the zoomed-in view describe the detailed flow structures. In the following sections, the processed images are spatio-temporally analysed based on the concentration, velocity and vorticity fields near the contact line of the evaporating sessile droplets.

### Stage I: Onset of the linear instability, non-linear growth and the micro-jet formation

**Figure 2:**
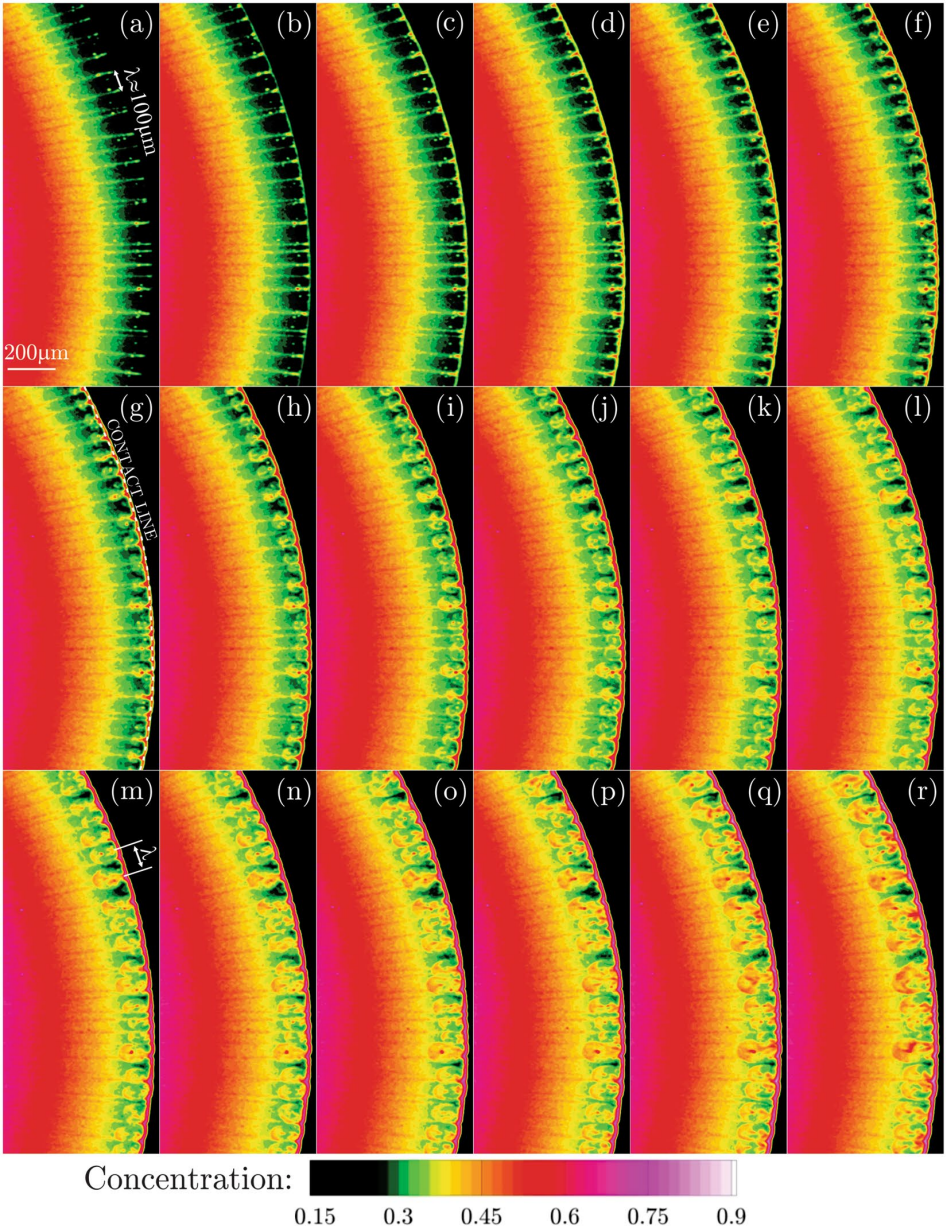
Spatio-temporal evolution of the concentration field of the nano-particles near the contact line of the evaporating sessile droplet with the time spacing of 140 *ms*. Stage I reveals the onset of the linear instability, non-linear growth and micro-jet formation. The streak lines aligned with the radius of the circular droplet characterize the initial wavelength of the instabilities estimated as *λ* = 100 *μm* as marked in the figure. (Supplementary Video Available).

In Fig. 2(a–r), the spatio-temporal concentration fields of the nano-particles inside the evaporating sessile droplet are presented. The images animate the instantaneous dynamics with the time spacing of 140 *ms*. In the panels (a–f) of the Fig. 2, some streak lines aligned with the radius of the circular droplet appear. In the azimuthal direction, these streak lines demonstrate high concentration regions separated by low concentration zones. Such a periodic high/low alternation of the concentration can be associated with some kind of instability to set in due to the growth of the perturbations. As identified in panel (a) of the Fig. 2, the initial wavelength associated with the instabilities can be approximated as *λ* = 100 *μm*.

It is important to describe the underlying physics resulting in the above instabilities by first discovering the source of the perturbations. Observation of the above high concentration streak lines separated by the low concentration regions suggests the action of a perturbation mechanism driving the nano-particles from the high pressure (low concentration) towards the low pressure (high concentration) streak lines. Now the question is to find the root for the pressure perturbations. According to the Marangoni type of the thermocapillary instabilities, any perturbation of the surface tension coefficient produces a Marangoni shear force (*F*_*M*_). The ratio of the *F*_*M*_ to the viscous diffusion force (*F*_*μ*_) defines the Marangoni number (*M*_*a*_). Accordingly, the Marangoni shear forces dominate the viscous forces for the large values of the Marangoni number and hence the instabilities set in. It is known that the evaporation flux near the contact line of the sessile droplets is azimuthally non-uniform resulting in the spatial temperature perturbations/gradients^29^. As a consequence of this non-uniform temperature field, the local surface tension coefficient also demonstrates corresponding perturbations/gradients. Lower surface tension occurs at the hot spots while the cold spots attain larger values of the surface tension coefficient. The Marangoni shear force is directed from the low surface tension (hot) spot to the high surface tension (cold) region. This induces a local flow velocity aligned with the direction of the shear vector. This is the so-called Marangoni flow which is correlated also with the direction of the pressure drop.

After the launch of the linear instabilities, the perturbations grow into a non-linear state. In the panels (g–r) of the Fig. 2, the line streaks demonstrate a spanwise thickening near the contact line. This non-linear phenomenon results in the formation of the micro-jet-like vortical structures ejecting from the droplet contact line and penetrating towards the center. In accordance with the radial penetration as wells as the spanwise growth of the micro-jet-like vortical structures, more nano-particles are entrained into the core of the structures. This entrainment is the consequence of the low pressure region induced due to the formation of the recirculation zones. The recirculation zones are here described qualitatively. The quantitative analysis of the micro-jet flow features are described in the concluding discussions of the results section in terms of the velocity and vorticity fields.

### Stage II: Micro-jet pairing

**Figure 3:**
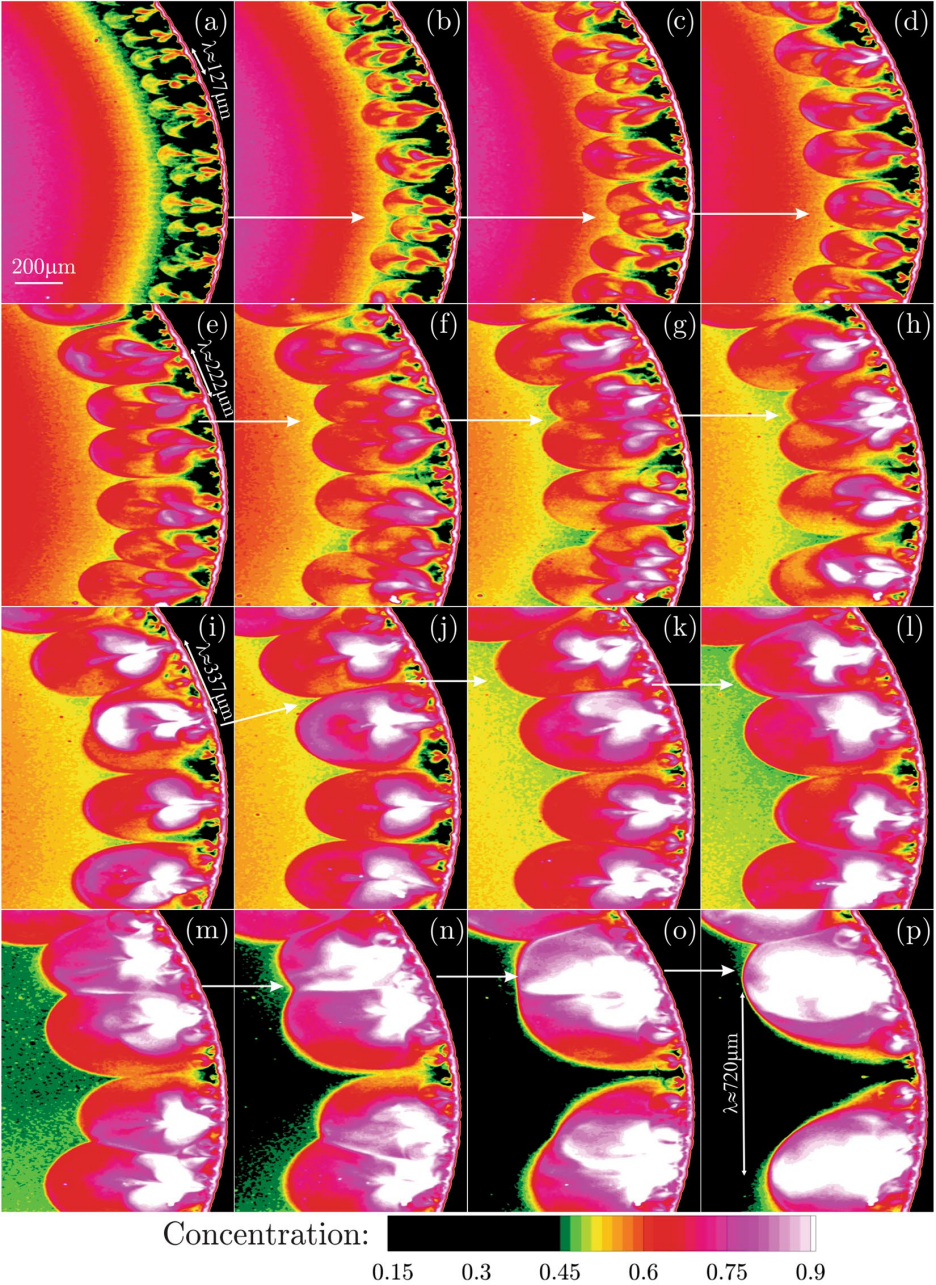
Spatio-temporal evolution of the concentration field of the nano-particles near the contact line of the evaporating sessile droplet with the time spacing of 13 × 140 *ms*. Stage II reveals the pairing of the micro-jet-like vortex structures of similar length scales. Due to the continuous occurrence of the pairing phenomenon, the initial wavelength of the instabilities grow as *λ* = 127, 222, 337, 720 *μm* as marked in the figure. (Supplementary Video Available).

As described above, the onset of the initially linear thermocapillary instabilities and the consequent growth of the perturbations into the non-linear state eventually results in the ejection of the micro-jet-like vortical structures from the contact line towards the droplet center. Starting from the time that the formation of the structures is rather accomplished, we focus on the mutual spatio-temporal interaction between the micro-jet-like vortices. In Fig. 3(a–p), concentration field of the nano-particles inside the evaporating sessile droplet is visualized near the contact line. Considering the panel (a) in Fig. 3 as the reference time instant, the following images are presented with the time spacing of Δ*t* = 13 × 140 *ms*.

As shown in the panel (a) of Fig. 3, the initial wavelength associated with the azimuthal spacing of the micro-jet-like structures is approximated as *λ* = 127 *μm* which is close to the earlier observation of the linear instabilities with *λ* = 100 *μm*. In the panels (a–d) of Fig. 3, the interaction of a pair of micro-jets are tracked in time followed by the direction of the arrows. It can be clearly seen that the pair of the micro-jets get closer until they merge eventually and form a larger jet-like vortex. Each of the jets entrain more particles into their low pressure recirculation core. In addition, the jet-like vortex structures grow in the spamwise direction due to the viscous diffusion effects. As a result, they become larger and start to interact with the neighbouring jet until the merging is accomplished. Consequently, the corresponding wavelength is almost doubled (the frequency reduces) to the estimated value of *λ* = 222 *μm* in Fig. 3(e). The recently formed paired vortices tend to coalesce again and form even larger structures as seen in the panels (e–i) of Fig. 3. Accordingly, the corresponding wavelength of the instabilities grows into *λ* = 337 *μm*. The pairing phenomenon continuously re-occurs as also portrayed in the panels (i–p) of Fig. 3 until the wavelength of *λ* = 720 *μm* is achieved in Fig. 3(p).

**Figure 4:**
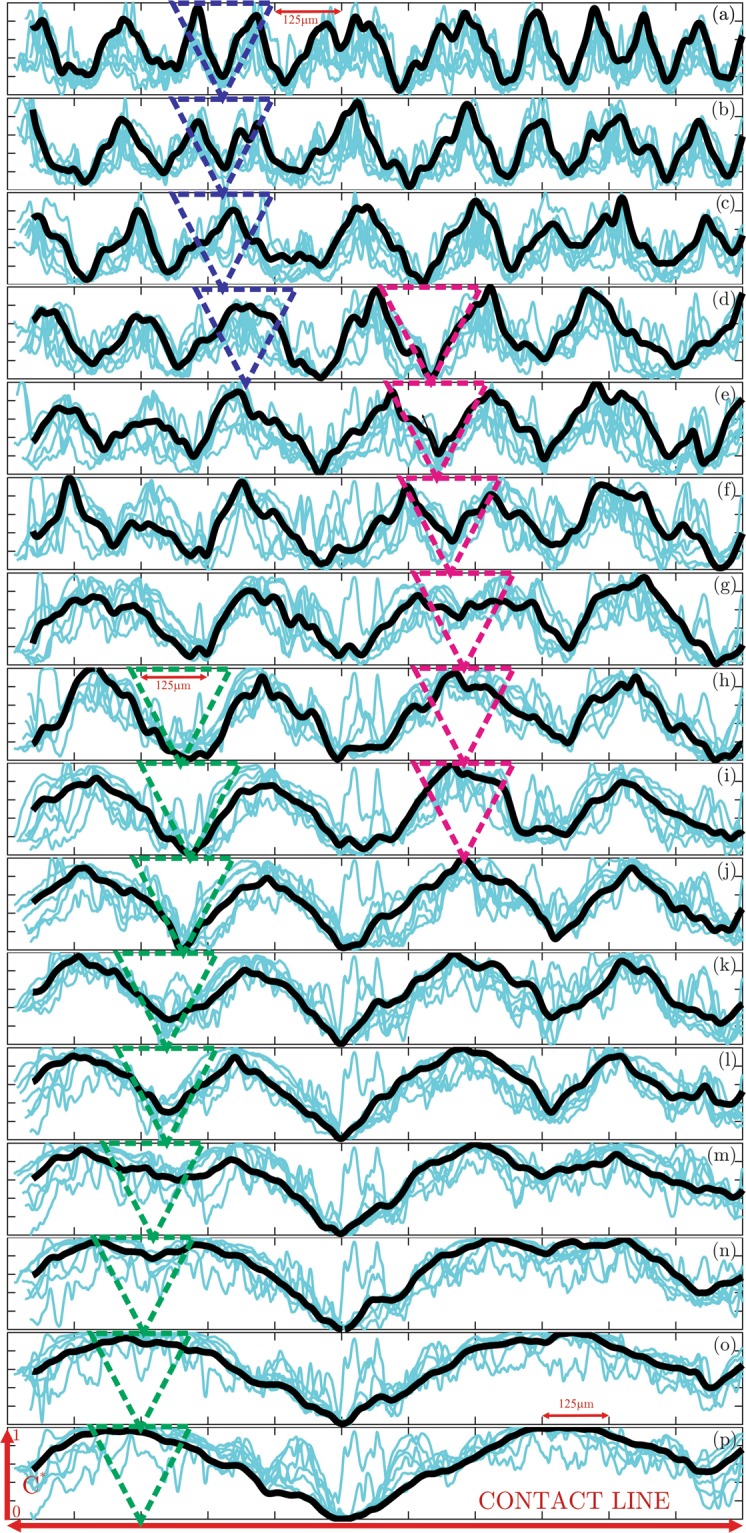
Profiles of the dimensionless concentration (C^*^) corresponding to the panels (a–p) of the Fig. 3 with the time spacing of 13 × 140 *ms*. The black line is the spatial average of instantaneous blue lines along the vortices.

In order to quantify the pairing process of the jet-like vortices, the temporal growth of the wavelength is characterized by the profiles of the dimensionless concentration (C^*^) and shown in Fig. 4. The line plots are corresponding to the panels (a–p) of the Fig. 3 with the same time spacing of Δ*t* = 13 × 140 *ms*. First, each field of view (FOV) associated with the slice of the droplet is mapped into the Cartesian coordinates where the contact line can be represented as the horizontal axis within each panel. Then, several line profiles of the concentration field (*C*) are extracted along the jet-like vortex structures. The instantaneous profiles extracted at different locations along the vortices are hard to interpret. This can be seen from the light blue lines shown in all panels. Therefore, they are interpolated within the same range to obtain the corresponding spatial average. For better comparison of the panels, the dimensionless concentration is defined as ${C}^{\ast }=\frac{C-{C}_{{\min }}}{{C}_{{\max }}-{C}_{{\min }}}$ ranging between 0 and 1 in the vertical axes. The spatially-averaged concentration profiles for each time instant (in panels (a–p)) are presented as the solid black lines. The profiles of the spatially-averaged dimensionless concentration nicely characterize the wavelength of the local vortex structures. Individual peaks in the profiles are associated with the micro jets while their span demonstrates the length scales. The micro jets at each time instant demonstrate rather similar length scales despite some variations.

With increased time, the jet-like vortices grow due to the viscous diffusion as well as entraining the ambient fluid and nano-particles. As a result, the wavelength profiles expand laterally. Some of the neighbouring jets start to get closer and eventually merge leading into larger structures. For clarity of the duration of the pairing, three different merging processes are marked using the dashed triangles. The first pairing (dark blue triangles) is observed in panels (a–d) with the time duration of 3Δ*t*. The second pairing (purple triangles) takes place during the time interval of 5Δ*t* as shown in panels (d–i). The third and the longest pairing (dark green triangles) process occurs within the time span of 8Δ*t* in panels (h–p). The interesting finding is the slow-down of the pairing process as the vortex structures and their corresponding wavelengths become larger. The jet-like vortex structures are decelerated as they grow larger due to the viscous diffusion and entrainment of more nano-particles. The resulting lower velocity and the larger merging distance result in the longer duration of the pairing. It should be also noted that the three pairing processes may occur with overlapping time intervals. It is revealed that the smaller scales (wavelengths) are associated with the high frequency (shorter time period) dynamics.

**Figure 5:**
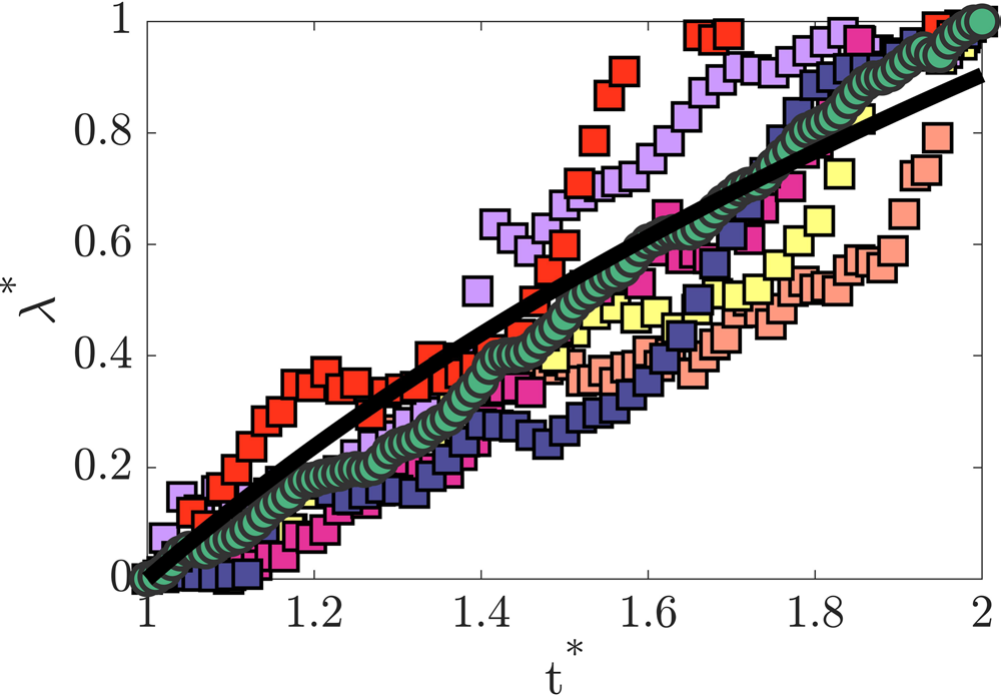
Temporal variation of the dimensionless wavelength (λ^*^). The square symbols of different colors represent measured data from 6 different drying droplets, and the average of these 6 sets of measurements is shown by the green circular symbols. The averaged data are fitted to the logarithmic profile of the form λ^*^ = *a* + *b*log(t^*^), and is shown by the black solid line.

Starting from the events corresponding to the time *t*_*a*_ (panel (a) in Fig. 3) to the time *t*_*p*_ (panel (p) in Fig. 3), the dimensionless time is defined as ${t}^{\ast }=1+\frac{t-{t}_{a}}{{t}_{p}-{t}_{a}}$. Similarly, the corresponding dimensionless dominant wavelength is described as ${\lambda }^{\ast }=\frac{\lambda -{\lambda }_{a}}{{\lambda }_{p}-{\lambda }_{a}}$ and presented in 5. The square symbols are associated with the measurements on six different evaporating droplets and show the increasing trend of the wavelength over time. These six different measurements on separate droplets are averaged (circular green symbols) to better demonstrate the statistics of the vortex evolution. Even though the initial wavelength growth shows a rather linear trend, the averaged values are fitted (solid line) to the logarithmic function of the form λ^*^ = *a* + *b*log(t^*^) to represent the following deceleration. The wavelength associated with the length-scale of the vortical structures is found to increase with the dimensionless time due to the viscous diffusion as well as the inverse cascade effects. While the early stage growth of the wavelength is observed from the ascending trend of the solid line, the growth rate is decelerated with increased time. This confirms the earlier observation in the concentration profiles. It might be mentioned that as seen in Figs 3 and 4 presented earlier, multiple wavelengths are present at each time instant, as might be expected^30^, because instabilities occur for a range of wavelengths and the instabilities occur in the present flow with a transient base flow leading to possibly changing dominant wavelength with time, and the measured phenomena are highly transient and never reach steady state until completely dried for the short drying process with complex flow field. This is also reflected in Fig. 5 where the temporal variation of the dominant dimensionless wavelength is shown for six different drying droplets, representative of the 47 droplets tested. It is seen that the dominant wavelength shows a similar and consistent increasing trend with time, but also exhibit some variations as expected.

### Stage III: Inverse energy cascade phenomenon

**Figure 6:**
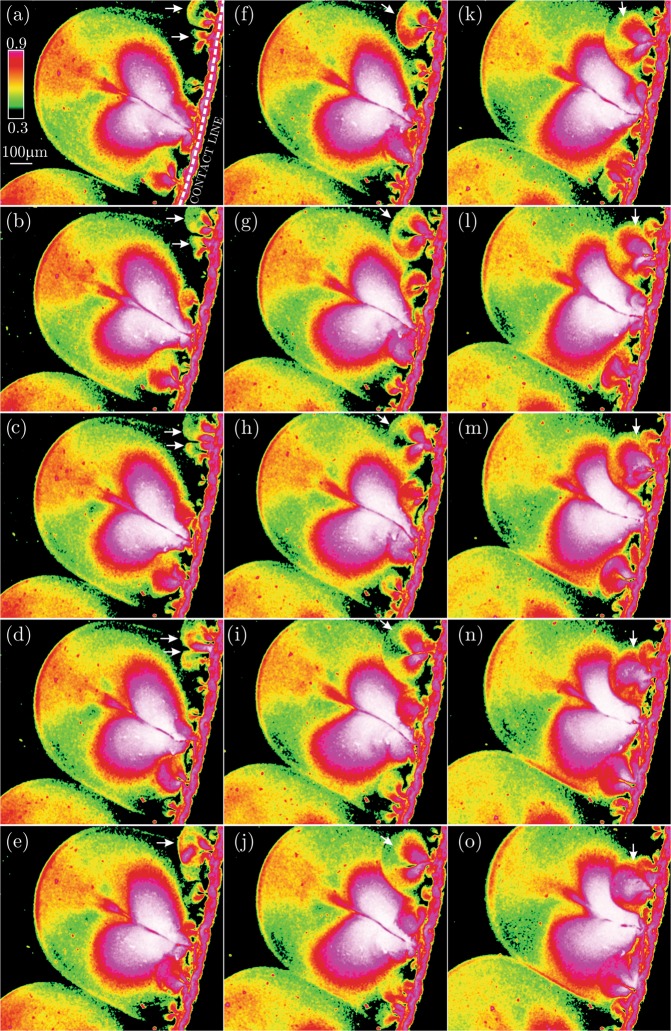
Spatio-temporal evolution of the concentration field of the nano-particles near the contact line of the evaporating sessile droplet with the time spacing of 2 × 140 *ms*. Stage III reveals two-types of the inverse cascade phenomenon. The first type is the pairing of the tiny jets to form larger vortex structures. The second type is the entrainment of the smaller jets into the larger jet-like vortices. (Supplementary Video Available).

Observing the pairing of the micro-jets with similar length scales in the previous section and the production of the larger vortical structures can be attributed to the first kind of the inverse cascade phenomenon where larger scales are continuously generated from the coalescence of the smaller vortices. In this section, a more interesting and a second type of the inverse cascade phenomenon is reported. To this end, a zoomed-in view of the larger structure jets obtained from the present microscopy are presented in Fig. 6. In Fig. 6(a–o), the concentration field is presented by the instantaneous images portrayed with the time separation of 2 *ms*.

At the first glance in every panel of Fig. 6, a large jet-like vortex is observed surrounded by the tiny neighbouring jets. The vortex pairing phenomenon described earlier, is also taking place for these tiny jets as clearly observed at the upper right corner of the panels (a–f) of Fig. 6. This zoomed-in view demonstrates that the tiny jets are continuously ejected from the contact line towards the center of the droplet while the large structures are growing. In the mean time, the nearby tiny jets near the largest jet-like vortex are entrained inward, or towards to the largest jet-like vortex. Particularly in the panel (g) of Fig. 6, different levels of the inverse cascade co-occur. While the vortex generated by the pairing of the tiny jets is entraining another tiny jet, the largest vortex is also absorbing other small scale vortices. Beyond this time instant, the arrows in the panels (g–o) of Fig. 6 clearly demonstrate the entrainment of the vortex generated from the pairing of the tiny jets into the largest jet-like vortical structure. The low pressure induced inside the larger jets keeps entraining the smaller scales inward. The spatio-temporal evolution of the vortical structures characterized by the present microscopy reveals the remarkable dynamics associated with the inverse cascade phenomenon. The inverse cascade is found to occur among micro-scale vortex structures of different sizes. One scenario is the pairing of the structures of the same length scale resulting in the formation of the largest structures. On the other hand, the smaller scales are continuously engulfed by the larger structures. Both of the mechanisms reveal the transfer of the kinetic energy from the smaller scales which are found responsible for feeding the larger vortices as they grow in space-time.

### Velocity and vorticity field of the micro-scale jet-like vortices

**Figure 7:**
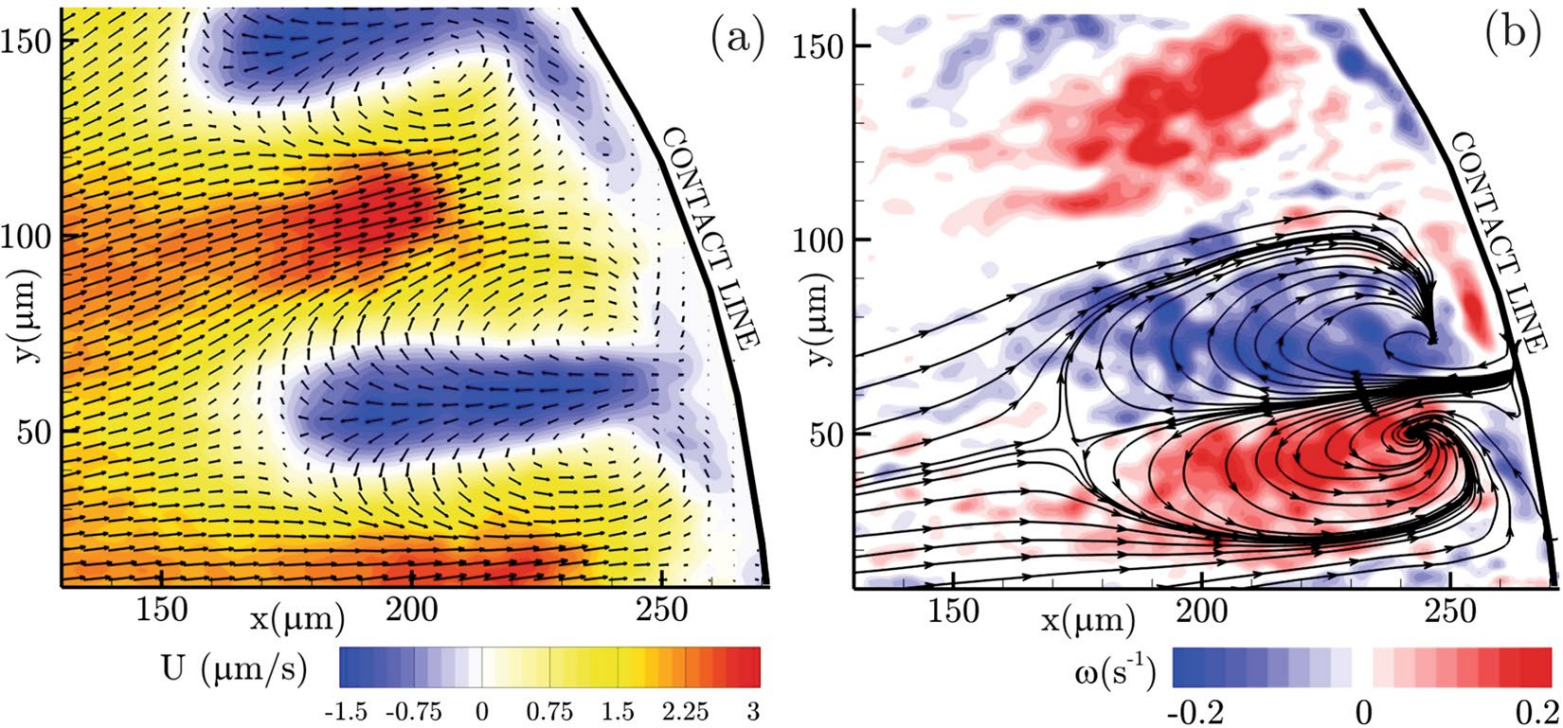
Time-averaged information obtained from the particle image velocimetry (PIV) for the zoomed-in view of the jet-like vortex structures near the contact line of the evaporating sessile droplet. (**a**) Velocity contour overlaid by the velocity vectors; (**b**) Vorticity fields superimposed with the velocity streamlines.

In Fig. 7, velocity and vorticity fields are calculated using the particle image velocimetry (PIV) analysis. Figure 7a demonstrates the time-averaged velocity contour overlaid by the velocity vectors. The pair of blue regions (negative velocity) characterize the jet-like structures flowing from the contact line towards the droplet center (right to left). On the other hand, there is a positive bulk flow from the center towards the contact line to account for the evaporated mass flux. Inside the jets, the velocity vectors diverge as the jet expands. It can be seen that the velocity vectors go around a center of rotation on either side of the jet.

To better characterize this recirculating flow region, the time-averaged vorticity field is calculated and presented in Fig. 7b. The vorticity contours are overlaid with the velocity streamlines for the clear depiction of the sense of rotation of the local flow elements. The streamlines and the colour of the vorticity field identify the pair of strongly counter-rotating recirculation zones. This is typically observed during the impulsive penetration of the jet flows. In addition, the tip of the present micro-jet-like vortex structure demonstrates a stagnation point where the positive and negative streamlines arrive and then deviate away. The stagnation point is typically a high pressure region formed at the tip of the micro-jets. For the present case, the stagnation point is formed at the tip of the micro-jet-like structures interacting with the radial bulk flow directed outward (from the center to the contact line). On the other hand, the recirculation zones on either side of the micro-jet-like vortex induce a low pressure field. In addition to the rotational flow on either side of the micro-jet-like vortex, the higher velocity gradients induce a shearing region characterized by the vorticity field.

## Discussion

Microscopic imaging as well as the particle image velocimetry (PIV) are conducted to obtain the concentration, velocity and vorticity fields near the contact line of the nano-particles-laden evaporating sessile droplets. The present study concerns the onset of the thermocapillary instabilities, formation of the micro-scale vortex structures with a focus on the remarkable observation of the inverse energy cascade phenomenon. The underlying mechanisms for the physical phenomena taking place near the contact line are explored in the three stages itemized below:

- Stage I: Onset of the linear instability, non-linear growth and micro-jet formation
- Stage II: Micro-jet pairing
- Stage III: Inverse cascade phenomenon

In stage I, streak lines of the high-concentration nano-particles are found to align with the radius of the circular droplet. At this stage of the onset of the linear instabilities, the streak lines portray azimuthally alternated high/low concentration zones with an estimated wavelength of *λ* = 100 *μm*. The emergence of such thermocapillary instabilities are linked with the Marangoni type surface tension gradients developed due to the perturbations in temperature/concentration. Accordingly, the perturbations translate into the pressure/velocity fields. Further growth of the instabilities into the non-linear state results in the formation of the jet-like micro-scale vortices ejected from the contact line towards the center.

In stage II, the individually ejected micro-jet-like vortical structures tend to grow in the spanwise direction due to the entrainment as well as the viscous diffusion. As a result, the neighbouring micro-jets start to interact and approach towards each other. Eventually, the pairs of the micro-jets coalesce and generate a larger structure. In accordance with the pairing of the vortices of the same size, the wavelength of the instabilities increase with time. In addition, the rate of pairing is found to reduce as the structures become larger due to the earlier merging. The continuous paring of the vortices of similar length scales and the creation of the larger structures is the first kind of the inverse mechanism observed in the present study. The second type of the inverse cascade phenomenon is observed in stage III where the smaller scale vortices are entrained into the larger ones. During this phenomenon, smaller vortices continuously feed momentum to the larger structures and contribute to their growth. It should be noted that the mechanisms described in the stages II and III may co-occur for variety of length scales.

Finally, the time-averaged velocity and vorticity fields are evaluated using the particle image velocimetry (PIV) analysis for the zoomed-in view of the large scale micro-jet structures. The velocity contour overlaid by the corresponding vectors characterize the jet-like vortex structures flowing from the contact line towards the droplet center as well as a bulk flow from the center towards the contact line to counterbalance the evaporated mass flux. Within the jets, the velocity vectors diverge as the jet expands while the velocity vectors go around a center of rotation near the edges of the jet. In addition, vorticity field overlaid with the velocity streamlines characterize the sense of rotation of the local flow. Furthermore, the tip of the present micro-jet-like vortex structures characterize a high pressure stagnation point while the pair of counter-rotating regions are formed on either side to induce a low pressure field.

## Methods

### Experiments

Droplets are placed on the horizontally levelled hydrophilic glass slides in the laboratory conditions of 294.7 K temperature and humidity levels of 70%. Back-light imaging technique is conducted by using the LED source emitting cold light (from below) to minimize its effect on the evaporation process. Optical microscopy is conducted for the instantaneous acquisition of the drying dynamics of the sessile droplets. Images of 1040 × 1388 pixels size are captured using the Carl Zeiss optical Microscope, Axio Zoom *V*16 with the magnification range of 0.7*X*–26*X* at the rate of 14 frames per second (fps).

### Ink and droplet preparation

Using a microliter syringe, catalyst ink droplets of the volume ~1 ± 0.01 *μL* are deposited on the surface. The catalyst ink consists of the *Pt*/*C* particles (HP 20% Platinum on Vulcan XC-72) and 5% wt Nafion solution (Ion Power D520, 45 ± 3% water, 50 ± 3% isopropyl alcohol, 5% NafionPFSA ionomer), with the isopropyl alcohol as the solvent. The solid component of the catalyst ink (Pt/C particles and Nafion polymer) is approximately at ~0.8 wt% in the solution where the Nafion to Pt/C weight ratios (*W*_*Nafion*_/*W*_*Nafion* + *Pt/C*_) are maintained within 25% to 50%. The ink mixture is treated by ultrasonic sonication to ensure fully dispersed particles and avoid agglomeration.

### Image analysis and velocimetry

The acquired images are processed to obtain the local concentration of the colloidal particles within the sessile droplet. The instantaneous variation of the concentration fields produce a map of the flow structures. In addition, LaVision’s DaVis 8 software is utilized to conduct particle image velocimetry (PIV) calculations from the captured images. The velocity field is evaluated by the cross-correlating pairs of successive images using an advanced multi-pass technique where the initial and final correlation passes of 64 × 64 pixels with 50% overlap and 24 × 24 pixels with 75% overlap are achieved. Using the universal outlier detection^31^, results are post-processed with the vector removal/replacement. Time-averaged flow-field is evaluated from the instantaneous information obtained at a rate of 7.14 Hz. The random errors associated with the PIV is analyzed based on the particle image disparity method^32^ which approximates the mean uncertainty of the velocity magnitude to be less than 10.8% within 95% confidence. The obtained uncertainty reasonably verifies the accuracy of the present velocimetry when compared to the typical values of 8–15% reported in the literature^33–35^.

## Supplementary information

Movie 1

Movie 2

Movie 3

## References

[1] Deegan RD (1997). Capillary flow as the cause of ring stains from dried liquid drops. Nature.

[2] Gomes G, Köberle R, Von Zuben CJ, Andrade DV (2018). Droplet bubbling evaporatively cools a blowfly. Sci. reports.

[3] Soltman D, Subramanian V (2008). Inkjet-printed line morphologies and temperature control of the coffee ring effect. Langmuir.

[4] Smalyukh II, Zribi OV, Butler JC, Lavrentovich OD, Wong GC (2006). Structure and dynamics of liquid crystalline pattern formation in drying droplets of dna. Phys. review letters.

[5] Brutin D, Sobac B, Loquet B, Sampol J (2011). Pattern formation in drying drops of blood. J. fluid mechanics.

[6] Kasyap T, Koch DL, Wu M (2014). Bacterial collective motion near the contact line of an evaporating sessile drop. Phys. Fluids.

[7] Mu X, Gray DG (2015). Droplets of cellulose nanocrystal suspensions on drying give iridescent 3-d “coffee-stain” rings. Cellulose.

[8] Bhar R, Kaur G, Mehta S (2018). Exploring drying pattern of a sessile droplet of genomic dna in the presence of hematite nanoparticles. Sci. reports.

[9] Yilbas BS (2018). Water droplet dynamics on a hydrophobic surface in relation to the self-cleaning of environmental dust. Sci. reports.

[10] Sobac B, Brutin D (2012). Thermocapillary instabilities in an evaporating drop deposited onto a heated substrate. Phys. fluids.

[11] Zhong X, Duan F (2017). Stable hydrothermal waves at steady state evaporating droplet surface. Sci. reports.

[12] Yunker PJ, Still T, Lohr MA, Yodh A (2011). Suppression of the coffee-ring effect by shape-dependent capillary interactions. Nature.

[13] Fukatani Y (2016). Effect of ambient temperature and relative humidity on interfacial temperature during early stages of drop evaporation. Phys. Rev. E.

[14] Al-Sharafi A, Yilbas BS, Ali H, AlAqeeli N (2018). A water droplet pinning and heat transfer characteristics on an inclined hydrophobic surface. Sci. reports.

[15] Wells GG (2018). Snap evaporation of droplets on smooth topographies. Nat. communications.

[16] Mampallil D (2015). Acoustic suppression of the coffee-ring effect. Soft matter.

[17] Yen TM (2018). Reversing coffee-ring effect by laser-induced differential evaporation. Sci. reports.

[18] Hoyas S, Fajardo P, Pérez-Quiles M (2016). Influence of geometrical parameters on the linear stability of a bénard-marangoni problem. Phys. Rev. E.

[19] Köllner, T., Schwarzenberger, K., Eckert, K. & Boeck, T. The eruptive regime of mass-transfer-driven rayleigh–marangoni convection. *J. Fluid Mech*. **791** (2016).

[20] Young RM, Read PL (2017). Forward and inverse kinetic energy cascades in jupiter’s turbulent weather layer. Nat. Phys..

[21] Chertkov M, Kolokolov I, Vergassola M (1998). Inverse versus direct cascades in turbulent advection. Phys. review letters.

[22] Kraichnan RH (1967). Inertial ranges in two-dimensional turbulence. The Phys. Fluids.

[23] Xia H, Byrne D, Falkovich G, Shats M (2011). Upscale energy transfer in thick turbulent fluid layers. Nat. Phys..

[24] Kolokolov, I. & Lebedev, V. Velocity statistics inside coherent vortices generated by the inverse cascade of 2-d turbulence. *J. Fluid Mech*. **809** (2016). 10.1103/PhysRevE.93.033104 27078444

[25] Xiao Z, Wan M, Chen S, Eyink G (2009). Physical mechanism of the inverse energy cascade of two-dimensional turbulence: a numerical investigation. J. Fluid Mech..

[26] Belmonte A (1999). Velocity fluctuations in a turbulent soap film: The third moment in two dimensions. Phys. Fluids.

[27] Shats M, Xia H, Punzmann H (2005). Spectral condensation of turbulence in plasmas and fluids and its role in low-to-high phase transitions in toroidal plasma. Phys. Rev. E.

[28] Seo SW, Ko B, Kim JH, Shin Y (2017). Observation of vortex-antivortex pairing in decaying 2d turbulence of a superfluid gas. Sci. reports.

[29] Karapetsas G, Matar OK, Valluri P, Sefiane K (2012). Convective rolls and hydrothermal waves in evaporating sessile drops. Langmuir.

[30] Drazin, P. G. & Reid, W. H. *Hydrodynamic stability* (Cambridge university press, 2004).

[31] Westerweel J, Scarano F (2005). Universal outlier detection for piv data. Exp. Fluids..

[32] Sciacchitano A (2015). Collaborative framework for piv uncertainty quantification: comparative assessment of methods. Meas. Sci. Technol..

[33] Bown M, MacInnes J, Allen R, Zimmerman W (2006). Three-dimensional, three-component velocity measurements using stereoscopic micro-piv and ptv. Meas. Sci. Technol..

[34] Lima R, Wada S, Tsubota K-i, Yamaguchi T (2006). Confocal micro-piv measurements of three-dimensional profiles of cell suspension flow in a square microchannel. Meas. Sci. Technol..

[35] Kinoshita H, Kaneda S, Fujii T, Oshima M (2007). Three-dimensional measurement and visualization of internal flow of a moving droplet using confocal micro-piv. Lab on a Chip.

